# Development of a pilot rare disease registry: a focus group study of initial steps towards the establishment of a rare disease ecosystem in Slovenia

**DOI:** 10.1186/s13023-019-1146-x

**Published:** 2019-07-09

**Authors:** Dalibor Stanimirovic, Eva Murko, Tadej Battelino, Urh Groselj

**Affiliations:** 1 National Institute of Public Health, Trubarjeva Street 2, 1000 Ljubljana, Slovenia; 2 Department of Endocrinology, Diabetes and Metabolism, UMC - University Children’s Hospital Ljubljana, 1000 Ljubljana, Slovenia; 3 Faculty of Medicine, University of Ljubljana, 1000 Ljubljana, Slovenia

**Keywords:** Rare diseases, Pilot rare disease registry, National rare disease registry, Rare disease ecosystem, Case study, Focus group, Slovenia

## Abstract

**Background:**

According to rough estimates, there are approximately 150,000 rare disease patients in Slovenia (out of a total population of 2 million). Despite the absence of accurate epidemiological data on their status, these figures reveal the great importance of this area for the Slovenian healthcare system. Consistent monitoring in the field of rare diseases facilitates evidence-informed healthcare policies, comprehensive observation of rare disease patients, and consequently serves increasingly demanding medical and statistical needs. This paper initially explores the current situation concerning rare diseases and identifies related challenges for the planned development of a national rare disease registry in Slovenia. Based on the research findings, the paper outlines the construction of the pilot rare disease registry and conceptualizes the establishment of a rare disease ecosystem in Slovenia.

**Methods:**

The research is based on a case study design, where focus group sessions were used as the main data collection technique. Structured discussions were conducted with 24 eminent experts affiliated with the leading institutions in the field of rare diseases in Slovenia. Analysis and interpretations of the data obtained were carried by means of conventional content analysis. A subsequent course of action for developing the pilot rare disease registry and conceptualizing the rare disease ecosystem was formulated in collaboration with the experts participating in the focus groups.

**Results:**

The research results indicate that the effective development of the national rare disease registry, followed by the establishment of the rare disease ecosystem in Slovenia, requires a broad approach that entails a whole series of systemic changes and considerations. Moreover, well-orchestrated and well-funded efforts to achieve this goal should involve the coordinated action of all stakeholders, including the amendment of the regulatory framework, quality design, and enactment of a general rare disease policy, as well as the alignment of medical, organizational, and technological aspects in accordance with the long-term public healthcare objectives.

**Conclusions:**

The establishment of a rare disease ecosystem in Slovenia and probably elsewhere, including a national rare disease registry, would represent an important improvement for patients, as it could significantly contribute to more coordinated healthcare treatment and enable comprehensive monitoring of the treatment process and results. A well-organized rare disease ecosystem could bring considerable benefits to healthcare system managers by providing a useful platform for estimating the required resources, evidence-informed policymaking, technological innovation, and organizational restructuring. This research provides valuable insight into the background of the issues that many countries face in the field of rare diseases, and ultimately provides practical recommendations for the development of national rare disease registries. However, ensuring effective healthcare delivery in this intricate field is critically dependent on the harmonization of digital solutions with other systemic factors and the adaptation of the rare disease ecosystem to the patients’ needs and the specifics of the healthcare environment.

## Background

According to rough estimates, there are approximately 150,000 rare disease (RD) patients in Slovenia [1, 2]. Despite the absence of accurate epidemiological data on the status of RDs, the above-mentioned numbers show that this area is of great importance for the Slovenian healthcare system. Besides the general lack of knowledge and resources in this field, RDs have specific and often intricate characteristics, which further increase the severity of this challenge for the healthcare system [3]. The problems in detecting and treating RDs include lengthy and arduous diagnostic procedures, often accompanied by difficulty choosing the most appropriate treatment method. The treatment approaches in the field are frequently underdeveloped and are often not supported by either empirical evidence (only trial treatments) or the existence of adequate medications. From the normative point of view, however, problems arise mainly from the necessity to provide an explicit legal basis for the monitoring of patients with RDs. Comprehensive monitoring of patients with RDs, coordination of their treatment, execution of screening, diagnosis, and meticulous reporting of identified new cases fall under the scope of organizational challenges, which often remain unresolved due to their inherent complexity and other systemic circumstances [2]. And then there is the information and communication technology (ICT) infrastructure, which should provide effective and user-friendly support to all stakeholders in the field of RD. Properly designed ICT solutions could be a very important tool for healthcare professionals in their clinical work with patients, for communication between stakeholders, as well as for healthcare management and the policymakers who direct the work of the entire healthcare system. However, regrettably, this field has been much neglected in terms of specialized ICT solutions and general ICT support.

RD registries in this sense represent one of the fundamental instruments of unbiased data collection, the monitoring of RDs, and epidemiological and clinical research, and can greatly contribute to improving healthcare planning and the treatment of patients [4]. Therefore, they are considered to be essential ICT components, as they aid in the collection, storage, analysis, and reporting of pertinent data on RDs to all stakeholders in the RD ecosystem. This ecosystem is considered to be a functional environment that incorporates all stakeholders and mechanisms for coordinated and comprehensive patient treatment. It should include the normative framework, the regulatory and policymaking bodies, clinical institutions for treatment and rehabilitation, patients and associations, ICT solutions (the national RD registry) and platforms (the national contact point for rare diseases; NCP), national and international RD organizations, statistical agencies and the research community, existing national and international data sources, and the precisely defined rules of operation and work processes between these entities [1]. As one of the main elements of the RD ecosystem, a RD registry in technological terms basically denotes a database of identifiable individuals containing a clearly defined set of health and demographic data collected for a specific public healthcare purpose [5]. Registration, on the other hand, could be defined as the process of the continuous systematic collection of data on the occurrence and characteristics of the related healthcare phenomenon. Moreover, RD registries facilitate comprehensive surveillance of the prevalence and incidence of RDs, and enable the well-founded evaluation of different aspects of healthcare procedures and outcomes [6]. Quality RD registries *ipso facto* provide a beneficial and applicable platform in all stages of evidence-informed healthcare policymaking, and may contribute to significant advancement in the management of RDs in general. For these reasons, the development of RD registries is one of the EU’s priorities in the field of the monitoring and control of RDs [7]. This is evidenced by specific recommendations and measures to support the development of such registries in different EU healthcare resolutions, strategic documents [8, 9], and EU-funded projects, including the RD-Connect (2012–2018) and EPIRARE (European Platform for Rare Disease Registries, 2011–2014) projects.

The RD-Connect project provided an integrated platform connecting databases, registries, biobanks, and clinical bioinformatics for RD research. This multidisciplinary project, which later gave rise to the RD-Connect Community, united partners from the EU and beyond to create an integrated global infrastructure in the field of RDs [10]. The EPIRARE project was focused on the conditions for the establishment of the EU registries and databases on RDs, legal issues, the definition of a common data set and procedures for quality control, and agreement on the registry scope, governance, and long-term sustainability thereof [11]. In view of these projects and other initiatives, RD registries in specific forms and of limited extent already exist in France, Spain, Italy, Slovakia, and Belgium, whereas those of Bulgaria and Sweden are in the process of being prepared [12]. The development and introduction of a RD registry in Slovenia is also one of the main concerns outlined in the “Work plan in the field of rare diseases in the Republic of Slovenia” from 2011 [1] and formulated in the “Resolution on the National Healthcare Plan 2016–2025” [13]. However, despite the unquestionable importance of the RD registry and initiatives from various international and supranational organizations, the legal basis for the establishment of the national RD registry was adopted only recently (19 April 2018). Consequently, Slovenia still does not have a functional RD registry. The RD registry was included in the proposed amendment to the Healthcare Databases Act [14], which was finally adopted after a long period of legislative gridlock.

In view thereof, the aim of this paper is to explore the existing state of affairs concerning RDs, outline the development of the pilot RD registry (PRDR), and propose the establishment of a RD ecosystem in Slovenia. Accordingly, this paper primarily focuses on the following interrelated research objectives:

- an analysis of the current situation and identification of related systemic constraints and deficiencies in the field of RDs in Slovenia;
- development of the PRDR and conceptualization of the RD ecosystem in Slovenia.

## Methods

### Research design

This paper employs a case study design to investigate the outlined research objectives concerning the current situation in the field of RDs, the development of the PRDR and conceptualization of the RD ecosystem in Slovenia. Fourteen focus group sessions were used as the main data collection technique and conducted from January 2016 to February 2017. The selection of the research method was based on the particularities of the research problem [15, 16]. Since this study was largely exploratory in nature, quantitative empirical methods could not yield satisfactory results or provide a credible assessment of the field. Namely, the complex field of RDs in Slovenia is still in an early developmental stage, and it would be difficult to ensure the representativeness of the research sample. For this reason, the focus group methodology was considered the most favourable methodological approach to carrying out an in-depth analysis of the situation, the identification of the necessary systemic changes, and shaping the preferred solutions in this domain.

### Sample

The selection of the focus group participants was primarily based on their experience and expertise in the area of RDs, which was intended to ensure the credibility of their views and facilitate constructive participation in the study. A non-random stratified sampling approach was used to ensure a representative sample of the healthcare experts that satisfied the required conditions. Assembling the focus groups was completed when the saturation point was reached. The final sample size comprised 24 experts, who were affiliated with different institutions: the National Institute of Public Health (NIPH) (3 participants), the University Medical Centre Ljubljana (UMCL) (11 participants), the University of Maribor Faculty of Medicine (6 participants), the Slovenj Gradec General Hospital (2 participants), and ICT companies (2 participants). The participants were qualified healthcare professionals specialized in microbiology and immunology, human reproduction, the cardiovascular system, biochemistry and molecular biology, oncology, metabolic and hormonal disorders, ICT experts in complex systems and cybernetics from the healthcare and private sector (consultants and analysts), and experts in public health (methodologists and statisticians).

### Data collection

The final goals of the focus group sessions were revised with the participants in line with their comments and suggestions, which helped to resolve some conceptual ambiguities. The participation rate in the focus groups was 100%; namely, all invited experts responded to the invitation and ultimately participated in the focus group sessions. The focus group sessions, which lasted approximately 90 to 120 min, were held on the premises of the UMCL. The directed and structured discussions were focused on the existing situation in the field of RDs in Slovenia and related problems, substantive, technological and organizational issues concerning the development of the PRDR, and priority actions at the normative and implementation levels. The participating experts had to identify and outline the priority areas and critical factors that they deem to be crucial for the successful development of the PRDR (and potential subsequent development of the national RD registry) and the establishment of the RD ecosystem. The discussions and responses of the focus group participants were recorded in writing.

### Data analysis

Analysis of the data obtained and the interpretation thereof were carried out by means of conventional content analysis [17, 18], while the platform for the development of the PRDR and the conceptualization of the RD ecosystem in Slovenia were derived from the focus group discussions and literature review. The content analysis was based on codifying the key statements related to each construct highlighted by the participating experts (priority areas and critical factors). The coding categories were derived from the focus group discussions. In order to increase the objectivity and credibility of the findings obtained, a final content analysis was carried out independently by several coders (the authors). Following the data analysis, the development of the PRDR and the subsequent conceptualization of the RD ecosystem were conducted in collaboration with the participating experts, who played a constructive role throughout all phases of the study. After an extensive review of the literature and investigation of primary and secondary sources containing RD-related content [1, 2, 4, 8, 9, 12, 13], the current situation concerning RDs in Slovenia was systematically analysed. In this phase, the research was especially focused on the imminent challenges in the field, in an effort to provide a feasible platform for the planned development of the PRDR and conceptualization of the RD ecosystem in Slovenia. The role of the participating experts within the proposed study was twofold. First, they had to participate in the analysis of the existing situation in the field of RDs. And second, drawing from their own experience and knowledge in the field, they had to provide their vision of the development of the PRDR (including its ensuing transformation into the operative national RD registry) and conceptualization of the RD ecosystem in Slovenia.

## Results

The experts participating in the focus groups outlined the state of affairs in the field and highlighted the priority areas and critical factors that need to be carefully considered in order to successfully implement the PRDR (and subsequently also the national RD registry) and establish a functional RD ecosystem (Table 1).

**Table 1:** Priority areas and critical factors in the RD field highlighted by the experts

Priority area	Critical factor
Clinical work	- Envisaged benefits and potentials of RD registries for clinical work with patients, designing standards, and research
	-Monitoring the effects of patient treatment and planning further procedures
	-Effective work and access to all relevant patient information in one place
Patient treatment perspective	- Better coordination and monitoring of the treatment process
	- Higher quality, safer medical treatment and better treatment results (better quality of life)
	-An effective communication channel and secure exchange of information
	- Evidence-based allocation of resources for patients with rare diseases
ICT infrastructure and solutions	- Effective and user-friendly ICT solutions (RD registry)
	- Ensuring interoperability despite the heterogenity of the information systems
	- Definition of the rules of operation, information flows, and organizational processes
	-The role of eHealth (the Central Registry of Patient Data (CRPD))
Normative framework	- Adequate legal basis
	-Protection of personal data (application of the safe-by-design principle)
	-Personal Data Protection Act / General Data Protection Regulation (GDPR)
Development of the PRDR and conceptualization of the RD ecosystem in Slovenia	- Strategic documents and development directions
	-Research and pilot projects
	-Adequate funding, engagement of stakeholders, and healthcare policy support
	-Selection of the appropriate approach for the development of the PRDR (and subsequently the national RD registry) and the establishment of the RD ecosystem in Slovenia

The listed priority areas and related critical factors (Table 1) were thoroughly discussed and scrutinized in the subsequent phases of the study. A general analysis of the current situation in the field, including the identified systemic constraints and deficiencies concerning the outlined priority areas and critical factors are presented in the following sections.

### Analysis of the current situation and identification of related systemic constraints and deficiencies in the field of RDs in Slovenia

#### Clinical work and the patient treatment perspective

The focus group participants initially identified and elaborated different issues regarding clinical work in the field and the patient treatment perspective. These two priority areas cover a large number of critical factors that are crucial for the overall situation in the field. The results presented below are derived from the focus group discussions.

For the classification and codification of RDs, healthcare professionals in Slovenia currently use the updated Australian modification of the 10th revision of the International Statistical Classification of Diseases and Related Health Problems (ICD-10-AM, version 6) proposed by the World Health Organization (WHO) [19]. Since there is only a narrow range of specific codes for around 6000 existing RDs contained in the ICD-10-AM, clinical practice in the field is rather diverse and inconsistent [2]. As legally required, the NIPH keeps records of the hospital treatment of patients whereby the main diagnoses and supplementary diagnoses are recorded [20]. The Institute of Oncology records the incidence of rare cancers in Slovenia as part of the Pan-European RARECARE project [21].

The Healthcare Databases Act, which represents the main legal basis, defines the types of databases and stipulates the conditions required for data processing [14]. Annex 1 to the Healthcare Databases Act defines the content of specific healthcare databases, their purpose, periodic reports, the manner of reporting, and data retention periods. The RD registry was only recently included in the Act. However, the Act had already contained five registries of diseases that are either rare or pertain to RDs. Of these five registries, which were previously listed in Annex 1 to the aforementioned Act, there are only three registries implemented in practice that include RDs, namely the Registry of Congenital Anomalies, the Registry of Children at Risk, and the Registry of Blood Clotting Disorders. In contrast, there are several clinical RD registries in Slovenia (Table 2) that lack legitimate status and an appropriate legal basis in the Act. Notwithstanding this, some of them already report data to the European registries (e.g. the Fabry’s Disease Registry, the Cystic Fibrosis Registry, etc.).

**Table 2:** The list of existing clinical RD registries

Registry name	
Registry of Patients with Cystic Fibrosis	
Registry of Patients with Inborn Errors of Metabolism	
National Registry of Patients with Fabry’s Disease	
Registry of People with Blood Clotting Disorders	
Registry of Patients with Renal Failure	
Registry of Patients with Neuromuscular Diseases	
Slovenian National Registry of Patients with Primary Immune Deficiency	
Registry of Congenital Anomalies	

Even though the RD registry was only recently included in the Healthcare Databases Act [14], it is rather obvious that the establishment of such a registry is an essential prerequisite for the comprehensive monitoring and management of RDs in Slovenia, and certainly represents one of the priorities in the field [22, 23]. Considering the needs of stakeholders in the field of RDs in Slovenia and international experience [12, 19], a well-designed and efficiently managed RD registry could provide many opportunities for improvements in the field, since it could be very instrumental in:

- monitoring prevalence and incidence and the consequent signalling of early warning signs;
- providing information for the development of suitable services at the national level and the development of suitable clinical pathways for the use of specialist services abroad;
- revealing the natural course of the disease – the characteristics of the disease, management, and outcomes with or without treatment;
- monitoring the safety of newly introduced or experimental treatments;
- evaluation of the clinical efficacy of new interventions;
- monitoring the results/outcomes of treatment and enabling a comparison with European and international standards;
- creating a list of patients that can be contacted for clinical trials or participation in multicentre studies;
- providing information on the economic evaluation of healthcare, such as disease costs and analyses of cost-effectiveness.

The coding of diseases by healthcare providers is currently adapted to the prevailing national practices and existing sectoral specifics. Subsequently, diagnoses of RDs can be coded according to different nomenclatures and terminologies, such as Orphanet, Online Mendelian Inheritance in Man (OMIM), or the Systematized Nomenclature of Medicine – Clinical Terms (SNOMED CT), depending on the context and the desired granularity of coding [24]. The 11th revision of the ICD with, thus far, approximately 5400 codes for RDs was released on 18 June 2018 [25, 26]. It is expected that this version with modifications will also be adopted by the Republic of Slovenia, but the process of adoption can take several years to complete. The data on hospital treatments is an important source of information for healthcare management and the assessment of the health status of the population. These sources of information are also used for reporting to the EU, the WHO, and other supranational and international organizations. In 2013, a uniform system for monitoring hospital treatments (named eTransfer) was implemented. This system significantly improves the monitoring of diagnosis-related groups (DRGs) and individual occurences within the DRGs [27]. Taking into account the planned adoption of the ICD-11, it should be considered whether the standard international coding of RDs should be implemented in all hospital information systems (the use of codes from the Orphanet nomenclature of RDs). Some countries (e.g. Germany and France) have already adapted the ICD-10 by applying extensions for the specific coding of RDs [19]. Furthermore, considering the anticipated requirements of the national RD registry, hospital information systems should certainly support the appropriate standardized coding of RDs and enable the transfer of healthcare data into the RD registry. Namely, patients with RDs are in most cases treated at four institutions in Slovenia – the UMCL, the Golnik University Clinic of Respiratory and Allergic Diseases, the Slovenj Gradec General Hospital, and the Valdoltra Orthopaedic Hospital. The rehabilitation of patients is carried out at the Soča University Rehabilitation Institute. The Centre for Undiagnosed Rare Diseases at the Clinical Institute of Medical Genetics of the UMCL is the first specialized unit for such diseases in the region [22].

#### ICT infrastructure and solutions

Concerning the broader field of ICT, the focus group participants pointed out various issues, ranging from typically technological to management (and policy) aspects, which concern the long-term planning of healthcare informatics, human resources, organization and operational processes, and sustainable funding. The presented findings are based on the focus group discussions.

When designing long-term ICT projects, an applicable model of management and sustainable funding must be considered in the early planning stages. It often happens in Slovenia that only start-up and pilot project funds are ensured in the planning stage. Only afterwards are questions about long-term management, the role of specific stakeholders, and the organization of their work often raised, although these issues should have been included in the development activities from the very beginning. This also relates to the provision of funds for ensuring the financial sustainability of the project, as well as maintaining, upgrading, and developing the ICT solution in the future. Ensuring suitable resources is a key prerequisite for the effective development of the national RD registry and its successful implementation in the complex healthcare environment. Moreover, the usefulness of the planned national RD registry will largely depend on the resources (financial, human, informational, organizational) available for its day-to-day management and continued operation. In accordance with the strategic documents for the regulation of this field and the establishment of the national RD registry in Slovenia, some important steps have been taken in the past few years. These are reflected in a more serious approach to this issue and the launching of the NCP for RDs. However, questions regarding the institutional management, vertical and horizontal organization, and long-term funding of the RD registry still remain largely unresolved. Since the implementation of the RD registry is one of the essential measures for successfully dealing with this important public health problem, the field of RDs is indeed not systemically regulated.

In addition, the ICT in the Slovenian healthcare is extremely heterogeneous, in the sense of both the different degrees of digitalization of particular healthcare services and the numerous different ICT solutions. Consequently, there have been many interoperability problems detected in the past, which partially still occur today, although to a much more limited extent. With the introduction of the eHealth solutions and the use of uniform infrastructure and data exchange standards, these problems have been largely eliminated. However, it must be emphasized that not all healthcare providers use the eHealth solutions and that some healthcare providers do not use them to their full extent. In the context of the planned establishment of the national RD registry, special consideration should be devoted to the Central Registry of Patient Data (CRPD). The CRPD contains a summary of patient healthcare data and patients’ medical records. The CRPD allows healthcare providers in Slovenia to access and exchange patient healthcare data to ensure high-quality patient treatment. For technical or other reasons, not all public healthcare providers in Slovenia send patient healthcare data to the CRPD, even though the central infrastructure has been operative for quite some time. Conventional healthcare data standards ensure a high level of interoperability and have improved the general quality of healthare data in the registries. In order to overcome the problems associated with interoperability between the local healthcare information systems and the national RD registry, the use of healthcare data standards must be planned in the early phases of the development of the RD registry. In addition, only adequately structured and standardized healthcare data can be transferred into the electronic records of the patients and subsequently used for various purposes (planning healthcare treatment, epidemiological and other public health studies, the preparation of public healthcare policies/programmes, etc.) [24].

#### Normative framework

The following results concerning the normative framework are largely based on a review of the documents (legal acts of the EU and the Republic of Slovenia). Only a small part of the results concerning the identification of neccesary changes in the Slovenian legal environment upon the enactment of General Data Protection Regulation is derived from the focus group discussions.

The valid Healthcare Databases Act provided a legal basis for the collection of data on patients with RDs only at the end of April 2018 [14]. The preparation of the amended Act was included in the regulatory work programme of the Government of the Republic of Slovenia for 2016, but since its adoption was not mandatory, all related activities were delayed [28]. However, based on the current momentum in the field of RDs and the tremendous efforts of the authors of this paper, the amended Act was adopted before the end of that term, namely in June 2018. The adoption of an appropriate Act is crucial to this end, as it provides the required legal basis for the collection of data on patients with RDs and the establishment of the national RD registry.

Directive 95/46/EC of the European Parliament and of the Council of 24 October 1995 on the protection of individuals with regard to the processing of personal data and on the free movement of such data provides the legal framework for personal data protection in the EU [29]. The Directive provides rules on the lawful processing and protection of personal data, and on the necessity of suitable control mechanisms concerning the protection of personal data. The Directive introduces the notion of individual consent and the prior notification of individuals regarding the processing of their data. In Slovenia, this area is regulated by the Personal Data Protection Act [30], which transposes the provisions of the Directive into the Slovenian legislation. On 25 January 2012, the European Commission published a proposal for the in-depth reform of the EU legislation regarding personal data protection. The aim of the reform was to protect personal data within the EU, increase citizens’ control over their own data, and decrease costs for companies. The new General Data Protection Regulation (GDPR) (EU) 2016/679 of the European Parliament and of the Council of 27 April 2016 on the protection of natural persons with regard to the processing of personal data, and repealing Directive 95/46/EC entered into force in May 2018 [31]. Since the Slovenian regulation and the Personal Data Protection Act [30] are very restrictive in terms of the protection of personal data, the introduction of specific systemic changes or other measures was not necessary. The only significant change was related to the establishment of a data protection officer in all organizations where sensitive personal data are processed. The legislation provides a binding framework that needs to be respected in the design, implementation, and use of the national RD registry. Furthermore, throughout the process of developing the national RD registry it is necessary to follow the software engineering principle of *safe by design*. This principle stipulates that special attention should be devoted to the safety of the product and the security of users during the entire planning, development, and implementation process.

### Development of the PRDR and conceptualization of the RD ecosystem in Slovenia

The results related to the development of the PRDR and the conceptualization of the RD ecosystem in Slovenia are generally derived from the focus groups and literature review. Each important paragraph in this section contains an explanation of which data the presented findings are based on.

Focus group participants outlined recent activities and projects in the field related to the potential development of the national RD registry. Unfortunately, an up-to-date strategic document and action plan in the field of RDs do not exist. Based on the strategic guidelines of the “Work plan in the field of rare diseases in the Republic of Slovenia” [1], the two-year research project “Analysis and development in the field of rare diseases in Slovenia” was launched in October 2015. The scope of the project includes a detailed sectoral analysis, the design of a national system for monitoring RDs, and the development of the PRDR [2]. In 2015, the Ministry of Health also launched a project for the creation of a website of the NCP for RDs, led by the Division of Paediatrics at the UMCL. The NCP was successfully established in 2016. The goal of the NCP is to create a network of stakeholders and to provide patients and experts access to high-quality information on the treatment of RDs is Slovenia [23]. The same stakeholders will provide the main data sources for the national RD registry upon its establishment in the future. The long-term goal, based on continuing to raise awareness of RDs, is to offer patients the option to self-register through the NCP.

Planning the development of the PRDR and conceptualizing the RD ecosystem was based on a distinctive methodological approach proposed by the focus group experts and already well-established formal steps from the literature [2]:

#### The registry’s main purposes

- The primary objectives of the PRDR were to address several of the traditionally unanswered issues regarding RDs that are important for the broader aim of improving the treatment of patients with RDs. Among these, we wanted to generate robust data on the incidence and prevalence of RDs in Slovenia, on the natural histories and diagnostic characteristics of RDs, and the management thereof at institutional and clinical levels. On that basis, we envisaged the PRDR as a means to improve not only the treatment of individual patients with a RD, but also to improve the RD ecosystem as a whole. Another important aim of the PRDR was also to ensure interoperability within the Slovenian eHealth system and to facilitate international data exchange.

#### Key stakeholders and the feasibility of the registry

- The PRDR was developed as a collaborative nationwide project by the institutions that are main stakeholders in the areas of RD clinical management, public health and epidemiology, and healthcare informatics. In addition, with the help of government representatives, we envisaged the appropriate regulatory framework for the national RD registry, including sufficient resources (i.e. we prepared a specific legislative proposal that was later incorporated into the Healthcare Databases Act [14]).

#### Registry team

- The PRDR preparatory team comprised an interdisciplinary group of professionals with backgrounds in clinical medicine, ICT/bioinformatics, genetics, and public health/epidemiology, thus representing all the main areas that need to be addressed in PRDR development and implementation. For practical implementation, it was also planned that the PRDR team would include administrators, coders, and an oversight body that includes patient representative(s).

#### Registry scope and data set

- It was proposed that the PRDR would include all patients diagnosed with a non-malignant RD and the pertaining OrphaCODE when diagnosed (all malignant RDs are subject to the national cancer registry). The proposed data set for the PRDR is presented in Table 3. For each patient, certain data sets (personal data, vital status) are to be generated from the Central Register of the Population of the Republic of Slovenia, while the remainder would be provided by the reporting physician on a standard reporting form. Importantly, the PRDR data set was additionally modified to ensure compatibility and interoperability with the recently proposed “Set of Common Data Elements for RD Registration” of the European Commission’s Joint Research Centre, in order to ensure international data exchange.

**Table 3:** Proposed data set for the Slovenian PRDR

Data set categories and their content	
1. Personal data: UMCN; name/surname; gender; place of birth	
2. Vital status: alive (Y/N); date and time of death	
3. Healthcare institution (of registration): name; department; date of first contact; date of registration; name of physician	
4. Diagnostic codes (main diagnosis): OrphaCODE; ICD-10 code	
5. Characteristics (main diagnosis): description of the diagnosis; date of diagnosis; confirmed (Y/N); age at diagnosis; time of first signs/symptoms (year/antenatal/at birth/ND)	
6. Other diagnoses (all to be listed): ICD-10 code; description	
7. Genetic characteristics and biological material: HGNC code; HGVS code; OMIM number; type of biological material available; biobank name	
8. Functionality/Disability scores: result according to the ICF classification	
9. Therapeutic data: any orphan drugs (according to the EMA list)	

List of abbreviations: *EMA* European medicines agency, *HGNC* The HUGO Gene Nomenclature Committee, *HGVS* Human Genome Variation Society, *ICD-10* International Classification of Diseases 10th Revision, *ICF* International classification of functioning, diseases and health, *N* No, *ND* Not determined, *OMIM* Online Mendelian Inheritance in Man, *UMCN* Unique Master Citizen Number, *Y* Yes

The technical/technological part of the PRDR development process was structurally based on recommendations from the literature [4, 11, 12, 32], and methodologically on the suggestions of the focus group participants. The whole technical/technological part of the project was carried out in parallel with the above steps and in accordance with the content outputs that were generated within each of the listed phases. The PRDR was designed as a web application that allows the competent subspecialized healthcare providers to report data on diagnosed RDs. Five reporting institutions collaborated on the project by participating in both the design of the PRDR itself and in its testing and final optimization. The construction of the PRDR was based on the open electronic health records (OpenEHR) methodology, which has become widely used to achieve semantic interoperability in healthcare and which was also used to create some analogous registries (e.g. the endoprosthetics registry) in Slovenia. This approach is “open” in terms of data, and the modelling and subsequent translation thereof into electronic form. The OpenEHR registry development methodology is based on the separate treatment of the clinical content and the ICT solution used for content management itself. The clinical content was planned, designed, and structured separately from the rest of the ICT solution development process. The point of this concept was that the clinical content preparation is done by clinical specialists, allowing the ICT analysts and developers to focus on the technical/technological aspects of the solution.

**Fig. 1:**
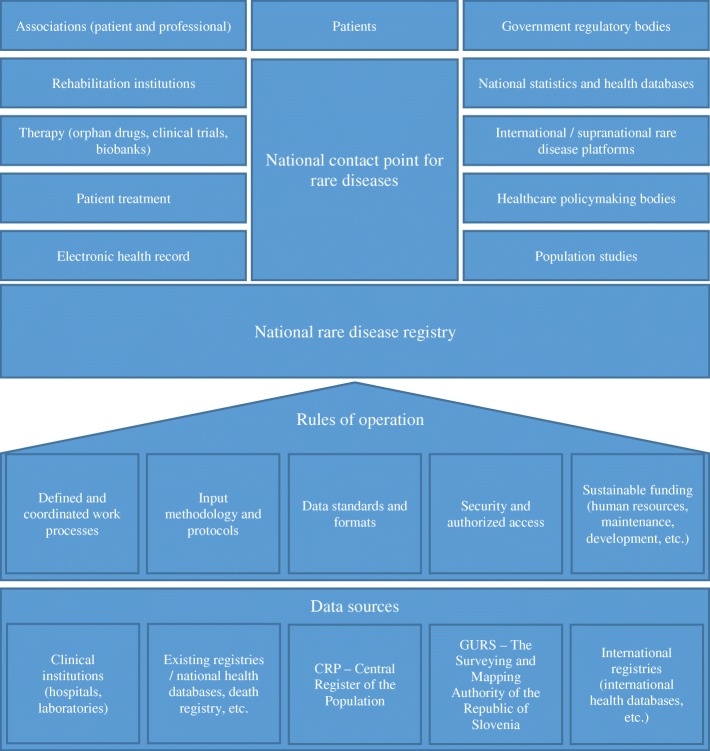
The proposed model of the RD ecosystem in Slovenia. Structure and organization of the proposed model of the RD ecosystem in Slovenia, including the national RD registry and all relevant entities in the field of RDs

Arising from the analysis of the current situation, priority areas, and identification of the critical factors in the field of RDs, we conceptualized the preferred RD ecosystem in Slovenia (Fig. 1) based on the recommendations of the focus group participants.

The national RD registry is envisaged as one of the central points of the RD ecosystem, providing a means for improving the treatment of patients with RDs. On the practical level, the national RD registry should be able to interconnect stakeholders to ensure the collection of relevant data (i.e. on RD incidence and prevalence, natural histories, and diagnostic characteristics, and their management at institutional and clinical levels), and also foster international data exchange. On the institutional level, the national RD should become a point of reference for RDs, systematically connecting the already existing but very fragmented infrastructure (e.g. the NCP for RDs, the existing RD registries), patients’ organizations, healthcare and academic institutions, and relevant governmental bodies. The reporting system for the national RD registry should be based on well-organized and reliable data sources, whereas standardized data transfers should be performed and controlled within the framework of precise and clearly defined rules of operation. In order to provide tangible benefits to all stakeholders, the analysis demonstrated that the operation of all entities in the RD ecosystem must be process- and outcome-oriented, as well as adequately technologically and normatively supported.

## Discussion

The literature reveals that national RD registries have complex and multidimensional effects on the entire field of RDs. There is general consensus that the construction of a comprehensive national RD registry presents one of the foundations for the systemic regulation of RDs in the country [33]. Based on the literature and focus group findings, a suitable RD registry could considerably contribute to more effective monitoring of RDs [34], improved patient treatments [32, 35], reduced inequality, and provide better support for evidence-informed policymaking [10, 11, 36]. In addition, based on the focus group findings, a well-coordinated RD ecosystem could bring considerable benefits to all healthcare managers, providing a useful platform for the estimation of the required resources, technological innovation, and organizational restructuring. However, it is clear that the implementation of an effective RD registry and the establishment of a comprehensive RD ecosystem require profound systemic changes and extensive efforts by the stakeholders, supported by targeted policy measures and sufficient funding.

This research provided an in-depth analysis of the current situation regarding RDs in Slovenia and enabled the identification of the most significant systemic constraints and deficiencies in the field. In addition, the paper presented a practical process for developing the PRDR and proposed a conceptual framework for the construction of the RD ecosystem. The systemic constraints and deficiencies in Slovenia identified by the focus group participants mainly concern the following matters:

- varying and inappropriate clinical practice in some parts of the coding process;
- regulatory shortcomings and non-compliance with legislation in the field of RDs;
- fragmented, non-interoperable, and inefficient ICT support;
- a lack of material and immaterial resources (financial, human, informational, organizational;
- institutional and project management, organizational, and operational process issues are still largely unsettled;
- the nonexistence of an up-to-date strategic document, action plan, evaluation framework, and well-defined measurable objectives (concerning the general issues in the field as well as the national RD registry and RD ecosystem);
- specific challenges related to the development, introduction, and utilization of the national RD registry and the RD ecosystem are difficult to predict at this stage.

The above-mentioned systemic constraints and deficiencies have an overarching impact on the priority areas and critical factors in the field of RDs highlighted by the focus group experts. Moving forward, these issues will have decisive implications for the successful future development of the RD registry and the construction of a functional RD ecosystem.

The development the PRDR has proven to be a very demanding task, since the entire process had to be conducted in a complex healthcare environment, and all developmental activities were critically dependant on the clinical, ICT, organizational, regulatory, and other important factors in the field of RDs. The forthcoming process of developing the national RD registry will have to be based on a feasible project plan that precisely defines the instutitonal organization and operational processes related to data flows. The material conditions for the management and long-term sustainability of the national RD registry will have to be ensured before the actual start of the project [36]. Similar principles will have to apply to the potential construction of the proposed RD ecosystem. However, due to its size and the number of entities involved, it will require even more effective cooperation, adherence to the rules, and the strong commitment of all stakeholders. The proposed model of the RD ecosystem does not seek to suggest a ‘one-size-fits-all’ solution to the numerous issues related to the establishment of the RD ecosystem. However, the presented study provides valuable insight into the background of RDs in Slovenia, and may provide the groundwork for further advances in this area.

### Practical recommendations

The experience of some EU countries and examples of good practice confirm that the successful establishment of the national RD registry demands a methodical development approach that includes the support of healthcare policies and the good collaboration of the stakeholders. Although there are no universal guidelines, some practical recommendations can be deduced from the literature [4, 5, 9, 11, 12, 35], which may be of assistance in similar projects focused on the development of national RD registries:

- Ensure political support from the highest level and establish an appropriate normative framework:
  - bring together all stakeholders and vendors from the private sector;
  - ensure the necessary funds and human and other resources;
  - prepare credible and viable strategy documents, feasibility studies, and action plans;
  - promote international collaboration and provide evidence-based projections for future benefits.
- Mobilize all stakeholders to ensure commitment and material and moral support, and to encourage their active participation and constructive criticism:
  - promote collaboration between policymakers, healthcare professionals, government officials, and ICT professionals;
  - provide an inclusive plan for communication within the project team and between the project team and all stakeholders.
- Establish an organizational and technological framework for the development of the RD registry:
- Define the main purpose of the registry, identify the key stakeholders and their assignments, organize an interdisciplinary team that consists of experienced individuals from the field, and define the registry scope and data set in accordance with your national needs and international guidelines;
- Choose a reliable and flexible technological platform that enables integration with other standardized information systems.

In addition to the recommendations from the literature, we formulated some more specific guidelines based on the focus groups in this study:

- Promote legislative amendments and adopt necessary regulations concerning the implementation of the RD registry and the establishment of the RD ecosystem.
- Establish a robust evaluation framework, including the objectives of evaluation, benchmarks and evaluation metrics, and define strategic and operative measures.
- Select a top manager and a quality project team with experience in complex ICT projects, and form a steering committee that includes diverse experts.
- Ensure adequate resources before the start of each project phase and make realistic plans in both temporal and financial terms, define milestones, and analyse operating and total costs.
- Perform constant supervision and strict control of already executed project tasks with respect to substantive and temporal objectives, and ensure the close monitoring of tasks that are in the execution phase.
- Improve or build a comprehensive ICT infrastructure (assess the current ICT infrastructure, interoperability issues, broadband connections, operating systems, network protocols, and data standards).
- Test the applicability of the RD registry in pilot projects and gradually implement individual ICT solutions in healthcare institutions:
  - promote the application of the RD registry;
  - organize education and training, issue standard practice guidelines.
- Inform stakeholders promptly and report all developments:
  - promote project achievements in order to improve and expedite the acceptance of the RD registry, facilitate comprehensive methodological explanations, create a user manual and help desk, and gain support from the media, experts, and citizens.

One should be aware that the listed recommendations depend on the current circumstances and several success factors, and cannot be easily transferred into practice. All these collaborative activities must be combined into functional and well-coordinated action, which is essentially the most challenging task of the project management team.

### Limitations of the study and future research directions

The research approach used in this paper has one clear methodological limitation. Since Slovenia does not yet have a national RD registry, the notions of the RD ecosystem including the national RD registry were hypothesized without concrete empirical validation in the actual healthcare environment. Accordingly, the issues related to the projected implications of the national RD registry and conceptualization of the RD ecosystem may raise some important questions of principle, while the research outcomes may therefore be disputable. These issues should be properly resolved in further research aimed at comprehensive analysis of the long-term effects caused by the establishment of the RD registry and the RD ecosystem. Future experiments should include detailed investigation of the applications and implications of the national RD registry, including its simulation and testing in the actual healthcare environment. The envisaged research goals should focus on facilitating the recommendations and operative guidelines for the establishment of such structures in all countries where patients are still not receiving adequate medical treatment due to systemic reasons and non-medical factors. Despite the outlined methodological limitation, the conducted research reveals the complex dynamics in the field of RDs in Slovenia, as well as the critical role of ICT in establishing a much-needed RD ecosystem, and is expected to contribute to theory building in the field.

## Conclusion

Ensuring more effective healthcare delivery in this specific field is strongly related to successful implementation of the national RD registry and its alignment with other systemic factors. The RD registry together with data sources, rules of operation, and NCP for RDs should form the ICT backbone of the RD ecosystem. The establishment of a comprehensive RD ecosystem, including the national RD registry, evidently requires the mobilization of all stakeholders, substantial funding, and the coordination of often conflicting interests within the healthcare system. This could present a major challenge for the effective long-term regulation of the field of RDs in Slovenia. However, due to recent developments, including the launch of the PRDR, and particularly the adoption of the umbrella law in this field, the overall situation concerning RDs looks more promising.

Notwithstanding the identified intricacies, the establishment of the RD ecosystem in Slovenia, including the national RD registry, undoubtedly represents a development opportunity that could efficiently connect different stakeholders, improve utilization of the already existing institutional capacities, and contribute to the improved healthcare treatment for all patients with RDs.

## Data Availability

Not applicable.
